# Redescription of *Setaria graberi* Shoho in Troncy, Graber & Thal, 1976 (Nematoda: Filarioidea) based on specimens from *Redunca arundinum* (Bovidae) in South Africa

**DOI:** 10.1051/parasite/2013042

**Published:** 2013-10-29

**Authors:** Ryno Watermeyer, John F. Putterill, Joop Boomker, Yuriy Kuzmin, Kerstin Junker

**Affiliations:** 1 Department of Veterinary Tropical Diseases, University of Pretoria Private Bag X04 Onderstepoort 0110 South Africa; 2 Agricultural Research Council-Onderstepoort Veterinary Institute, Parasites, Vectors and Vector-borne Diseases Programme Private Bag X05 Onderstepoort 0110 South Africa; 3 Department of Zoology, University of Johannesburg PO Box 524 Auckland Park 2006 South Africa; 4 Department of Parasitology, Institute of Zoology 15 Bogdan Khmelnytskyi Street Kyiv 01601 Ukraine

**Keywords:** Helminth parasites, Filarioidea, Onchocercidae, *Setaria graberi*, *Redunca arundinum*, South Africa

## Abstract

The filarial onchocercid *Setaria graberi* Shoho in Troncy, Graber & Thal, 1976 is redescribed from the abdominal cavity of Southern reedbuck, *Redunca arundinum* (Boddaert), in South Africa, including illustrations and scanning electron micrographs of important morphological features. Morphometric data for this species are provided for the first time. *Setaria graberi* is characterised by the possession of bifid deirids, and females having a distinctly bulbous tail. The slightly raised peribuccal crown forms a dumbbell-shaped unit with the cephalic elevations in apical view; the dorsal and ventral elevations, spaced 73–115 μm apart in females and 71–93 μm in males, carry two well-separated tips each. In dorsoventral view, the cephalic elevations appear more or less rectangular with a slightly notched apex and are narrow in comparison to the width of the anterior end. They are triangular in lateral view. Four cephalic and four external labial papillae are arranged in a laterally elongated rectangle each. The species is distinguished from other *Setaria* Viborg, 1795 species that possess bifid deirids or occur in members of the same host genus. The presence of *S. graberi* in *R. arundinum* in South Africa constitutes a new host and geographic record.

## Abstract (fr)

La filaire Onchocercidae *Setaria graberi* Shoho in Troncy, Graber & Thal, 1976 est redécrite à partir de spécimens de la cavité abdominale du Grand Cobe des roseaux, *Redunca arundinum* (Boddaert), en Afrique du Sud, en incluant des illustrations et des images en microscopie électronique à balayage des aspects morphologiques importants. Des données morphométriques sont fournies pour la première fois pour cette espèce. *Setaria graberi* est caractérisée par la possession de deirides bifides, et par des femelles ayant une queue distinctement bulbeuse. La couronne péribuccale légèrement élevée forme une unité en forme de cloche avec les élévations céphaliques en vue apicale ; les élévations dorsales et ventrales, séparées par 73–115 μm chez la femelle et 71–93 μm chez le mâle, portent chacune deux extrémités bien séparées. En vue dorso-ventrale, les élévations céphaliques apparaissent plus ou moins rectangulaires avec un apex légèrement entaillé et sont étroites par rapport à l’épaisseur de l’extrémité antérieure. Elles sont triangulaires en vue latérale. Quatre papilles céphaliques et quatre papilles labiales externes sont arrangées en des rectangles allongés. L’espèce est distinguée des autres espèces de *Setaria* Viborg, 1795 qui possède des deirides bifides ou se trouvent chez des hôtes membres du même genre. La présence de *S. graberi* chez *R. arundinum* en Afrique du Sud représente un nouvel hôte et une nouvelle mention géographique.

## Introduction

While studying the parasite fauna of artiodactylids in Tanzania between 1964 and 1967, Sachs & Sachs [9] recovered onchocercid specimens belonging to the genus *Setaria* Viborg, 1795 from the abdominal cavity of a number of hosts, including Common reedbuck, *Redunca redunca* (Pallas). These specimens were forwarded to Dr. C Shoho, then Animal Health Officer of the FAO in Somalia, for identification. Subsequently, Shoho in 1967 informed his colleagues in a personal communication (quoted by Sachs & Sachs [9]) that the material from *R. redunca* contained two *Setaria* species, *S. boulengeri* Thwaite, 1927 and a yet undescribed species, provisionally named *S. graberi*. At the time, the description of *S. graberi* was held back, as Shoho was in the process of preparing a revision of the genus *Setaria* in African hosts (Shoho, personal communication 2000). Shoho’s intention was to publish this manuscript, including the description of *S. graberi* as a new species, in collaboration with Dr. Sachs at the Tropical Institute in Hamburg. The latter, however, left the institute and the manuscript was never published. A decade later, Shoho made his unpublished manuscript entitled “*Setaria* worms from wild animals, mostly ruminants, of Eastern and Southern Africa and some other filarid worms” available to Troncy et al. [11], when these authors examined material from *R. redunca nigeriensis* Blaine in the Central African Republic and the Republic of Chad. Troncy et al. [11] accepted *S. graberi* as described by Shoho as a valid species and assigned their specimens to this taxon, listing Shoho as author. In the absence of a published description, Troncy et al. [11] provided detailed drawings of their specimens of *S. graberi* and stated that their material corresponded well with the illustrations provided in Shoho’s manuscript.

Through the kind facilitation of Prof. S. Uni, Osaka City University, Japan, Dr. Shoho forwarded parts of his unpublished manuscript that dealt with the “Group of *Setaria* worms from the Reedbucks, *Redunca arundinum* Boddaert and Bohor Reedbuck, *Redunca redunca bohor* Pall.” to the present authors. According to this part of the manuscript, which does not include any illustrations, the material of *S. graberi* studied by Shoho comprised one male and one female specimen from Southern reedbuck, *R. arundinum* (Boddaert), from Pilgrimsrest, Mpumalanga Province (then Transvaal), South Africa, which had been on loan from the National Collection of Animal Helminths (formerly the Onderstepoort Helminthological Collection), and six females and an unspecified number of males from *R. redunca* from Lake Rukwa, Tanzania; the latter originating from the collection of Sachs & Sachs [9]. Measurements were based on one male and one female from *R. redunca*, and “type material (was) preserved at the London School of Hygiene and Tropical Medicine”. Attempts to retrieve these specimens failed [Mrs. E. Harris, The Natural History Museum, London (NHM), personal communication 2004 and Dr. L. Gibbons, formerly NHM, personal communication 2004].

In this paper, we describe and illustrate *S. graberi* from material collected by Boomker et al. [1] from *R. arundinum* in KwaZulu-Natal, South Africa. Re-examination of said material showed that, in addition to *S. bicoronata* (Linstow, 1901) Railliet & Henry, 1911 and females of *Setaria* sp. recorded by Boomker et al. [1], *S. graberi* was also present. Scanning electron micrographs of important diagnostic characters are provided. We conclude that *S. graberi* Shoho in Troncy, Graber & Thal, 1976 is a valid species that can morphometrically be distinguished from all other members of the genus. The presence of *S. graberi* in *R. arundinum* in South Africa constitutes a new host and geographic record.

## Materials and methods

Specimens examined during the current study were collected and stored in 70% ethanol. They had been obtained during a previous study by Boomker et al. [1], a survey of the helminth community of *R. arundinum* in the Eastern Shores Nature Reserve (ESNR), KwaZulu-Natal, South Africa, from 1983 to 1984. The ESNR is situated at the southern end of the Mozambique coastal plain, between 27° 51′ and 28° 25′ S latitude and 32° 20′ and 32° 40′ E longitude [5].

Body length was determined by gently straightening specimens on a ruler and taking the reading using a stereomicroscope. Subsequently, nematodes were cleared in lactophenol and examined using a compound microscope (Olympus BX51) equipped with a drawing tube and digital camera (Olympus DP72). Measurements were taken with the aid of digital imaging software (Olympus cellSens Dimension, version 1.4.1). Drawings were made with the aid of the drawing tube. Microfilariae were dissected from the uterus of one female at the level of the oesophago-intestinal junction and cleared in lactophenol. Specimens for scanning electron microscopy (SEM) were dehydrated through a graded ethanol series and critical point-dried from absolute ethanol. Subsequently, head and tail segments were mounted individually onto conical brass SEM viewing stubs and sputter coated with gold. Samples were viewed and micrographed using a Hitachi S-2500 scanning electron microscope at an accelerating voltage of 8 kV.

Measurements are given as the range, with the mean and the number of measurements taken (*n*) in parentheses; when available, this number is followed by measurements of a single male and female each from *R. r. bohor* from Tanzania, described in Shoho’s unpublished manuscript (Shoho, personal communication 2000). Measurements are in micrometres, *n* = 8 for males and *n* = 9 for females, except when otherwise indicated. Male caudal papillae are numbered following Chabaud & Petter [2].

## Results

### *Setaria graberi* Shoho in Troncy, Graber & Thal, 1976

*Host*: *Redunca arundinum* (Boddaert) (Bovidae).

*Locality*: Eastern Shores Nature Reserve, KwaZulu-Natal, South Africa, situated between 27° 51′ and 28° 25′ S latitude and 32° 20′ and 32° 40′ E longitude.

*Collection date*: May 1983 to May 1984.

*Site of infection*: Abdominal cavity.

*Material deposited*: The specimens have been deposited in the collection of the Muséum National d’Histoire Naturelle, Paris, France, MNHN HEL358 (five males, five females) and the National Collection of Animal Helminths, ARC-Onderstepoort Veterinary Institute, South Africa, NCAH/S/2013/2 (three males, four females).

*Prevalence and intensity*: In 18 out of 22 (82%) host individuals that were re-examined, with intensity of infection ranging from 1 to 17 and a mean of 4.3 (±4.3).

### Description (Figures 1–4)

**Figure 1.**
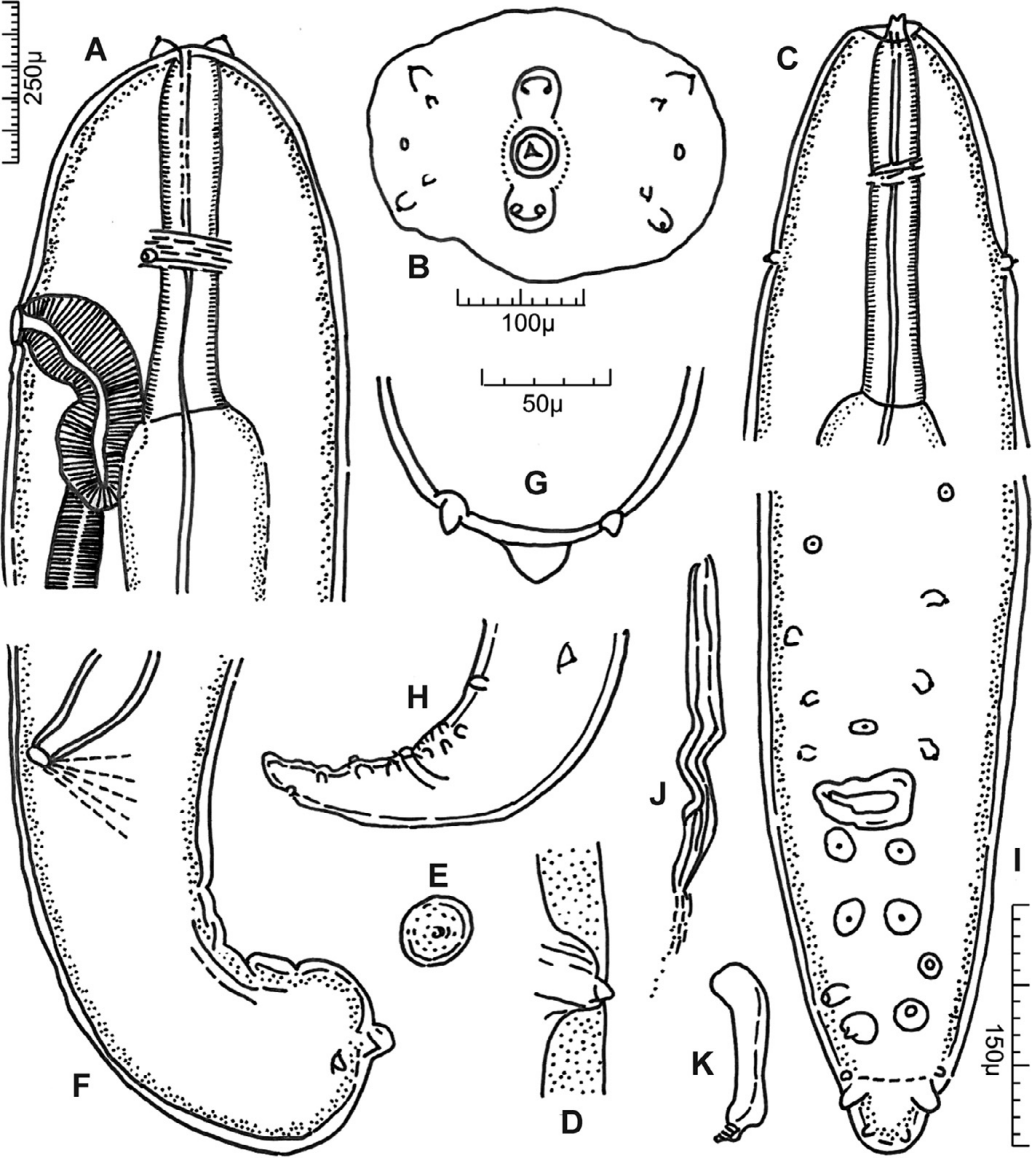
*Setaria graberi* Shoho, re-drawn after Troncy et al. (1976). A, female anterior extremity, lateral view. B, apical view. C, male anterior extremity, dorsoventral view. D, E, deirids, lateral and apical view, respectively. F, female posterior extremity. G, female, caudal tip. H, male, caudal extremity, left lateral view. I, male, caudal tip. J, K, left and right spicule, respectively. Scales in μm: A, C, H, 250; B, F, J, K, 100; D, E, 30; G, 50; I, 150.

**Figure 2.**
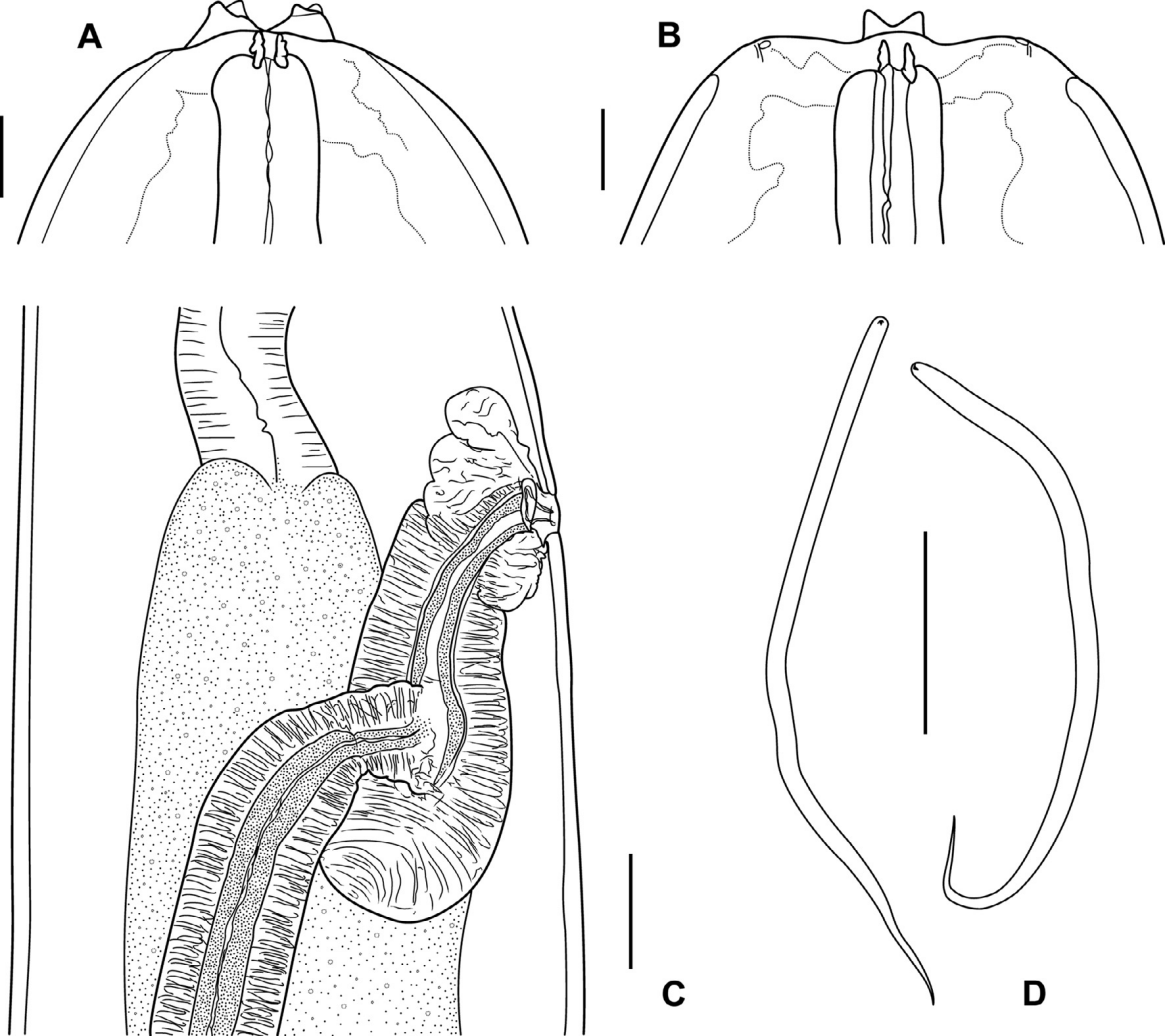
*Setaria graberi* Shoho. A, head, female, lateral view. B, head, female, ventral view. C, vagina and ovejector, female, lateral view. D, microfilariae. Scales in μm: A, B, D, 50; C, 100.

Long and slender filarial nematodes, off-white in colour. Four cephalic papillae and four external labial papillae arranged in laterally elongated rectangle each (Figure 4C); amphids approximately on mid-level between cephalic and external labial papillae (Figures 1B and 4C). Oral opening small, oval to round. Peribuccal crown slightly raised, forming dumbbell-shaped unit with cephalic elevations in apical view (Figures 1B and 4C); circular base of dorsal and ventral elevations tapering towards narrow midsection that surrounds oral opening; long axis dorsoventrally orientated (Figure 1B). Both dorsal and ventral elevations drawn out into two, well-separated, stubby tips in apical view (Figure 4C); elevations stump-like in median view, with narrow base in relation to width of head, more or less parallel sides and a notched apex (Figures 1C, 2B and 4B), triangular in lateral view (Figures 1A, 2A and 4A). Deirids at level of posterior half of muscular oesophagus, in females usually close to level of vulva (Figure 1C); bifid, situated on small, rugous, slightly inflated promontories; the two projections elongated, conical, with rounded tips, one slightly shorter, the difference in length more or less pronounced depending on specimen orientation, but most distinct in apical view (Figure 4D–F). Excretory pore not seen.

**Figure 3.**
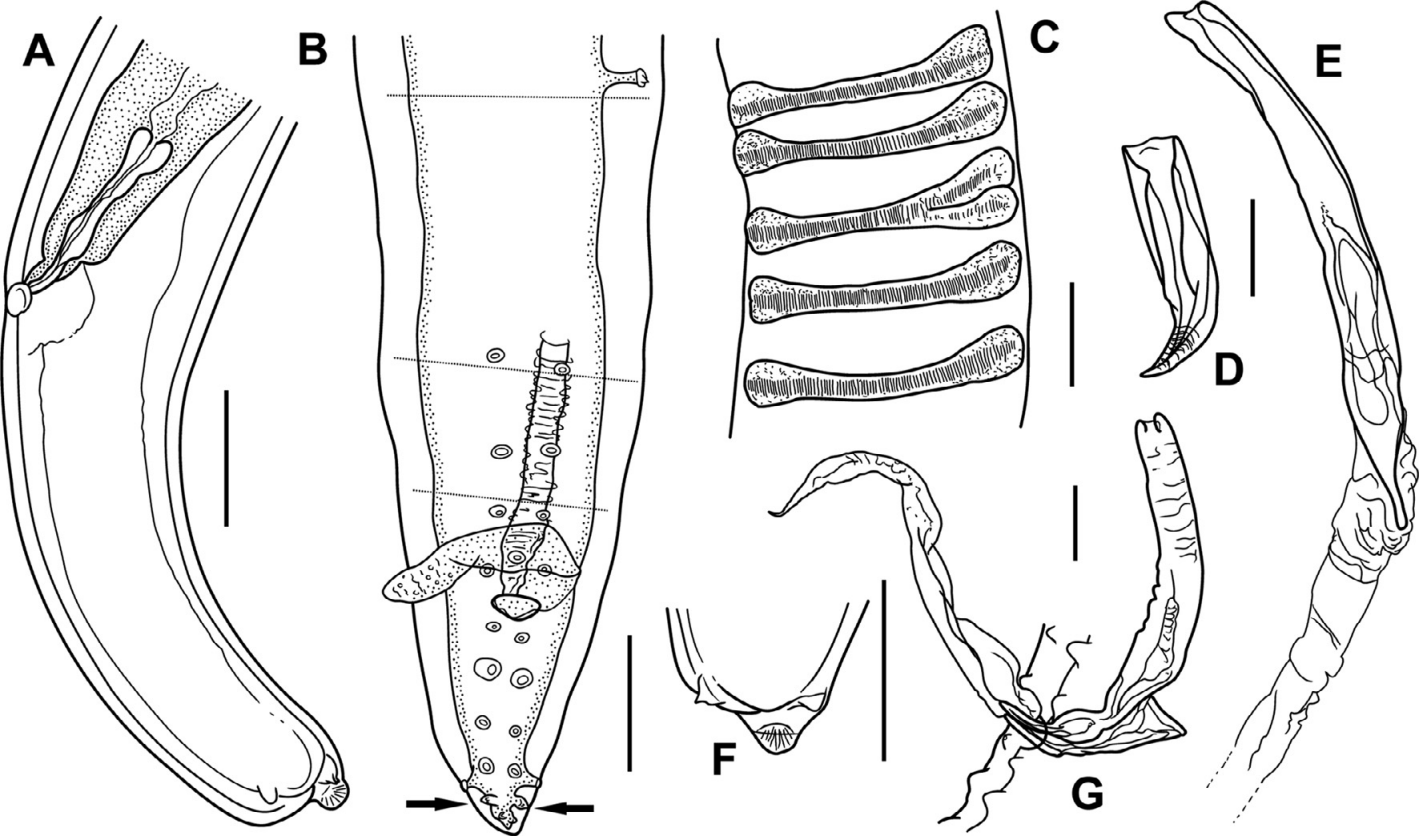
*Setaria graberi* Shoho. A, female, posterior extremity, lateral view. B, male, posterior extremity, ventral view, illustrating arrangement of left postdeirid and caudal papillae. Lateral appendages indicated by arrows. *Area rugosa* and right spicule not illustrated; horizontal lines indicating folds resulting from coiling. C, detail of *area rugosa* of male, ventral view. D, right spicule, ventrolateral view. E, left spicule, ventrolateral view, end of membranous sheath not shown. F, tip of tail, female, ventral view. G, left and right spicule of male, lateral view, membranous sheath of left spicule protruding from cloaca. Scales in μm: A–C, F, 100; D, E, G, 50.

**Figure 4.**
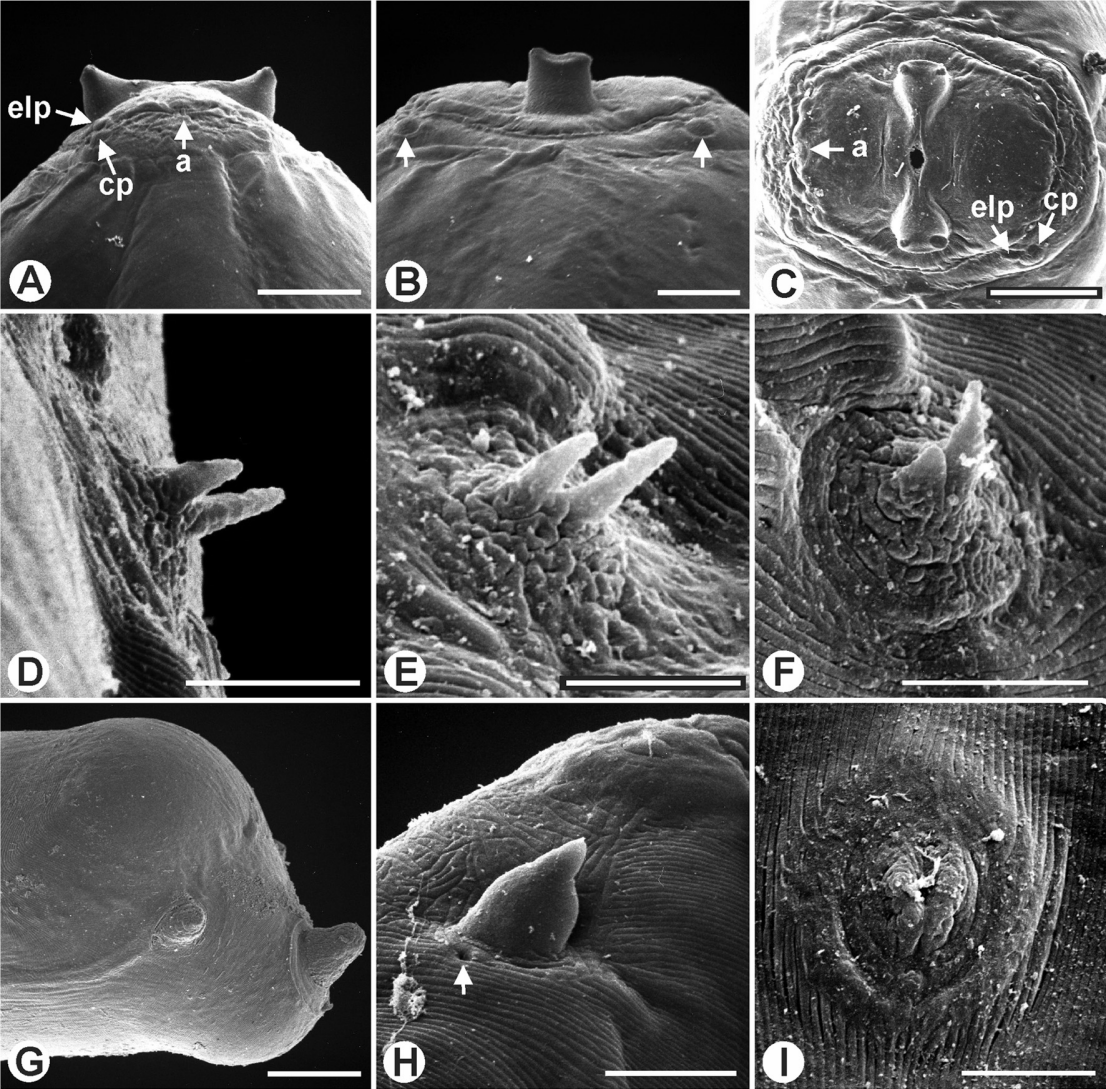
*Setaria graberi* Shoho, scanning electron micrographs. A–C, cephalic elevations, female. A, lateral view; a, amphid; cp, cephalic papilla; elp, external labial papilla. B, dorsoventral view; arrows indicating cephalic papillae. C, apical view; a, amphid; cp, cephalic papilla; elp, external labial papilla. D–F, deirids, female. D, E, ventrolateral view. F, apical view. G, posterior extremity of female. H, lateral appendage of male posterior extremity; arrow indicating phasmid. I, postdeirid of male. Scales in μm: A–C, 50; D, E, 5; F, H, I, 10; G, 20.

*Male*: Body length 46–50 (48; 42) mm. Maximum body width at level of oesophago-intestinal junction 372–560 (435, *n* = 7; 437 maximum width). Cephalic elevations 71–93 (81, *n* = 7; 74) apart in lateral view; distance between tips of cephalic elevations in dorsoventral view 23–31 (27, *n* = 7). Buccal capsule small and inconspicuous, 19–24 (21, *n* = 7) long and 16–21 (19, *n* = 7) wide in lateral view, 18–23 (20, *n* = 6) long and 17–24 (20, *n* = 6) wide in dorsoventral view. Nerve ring and deirids at 209–284 (231, *n* = 7; 234) and 396–485 (434, *n* = 7; 297) from anterior end, respectively. Muscular oesophagus 375–549 (461, *n* = 7; 500) long; often bent and folded as result of muscle contraction. Glandular oesophagus 4.2–9.1 (6.7, *n* = 6; 7.0) mm long. Oesophagus total length 4.6–9.6 (7.1, *n* = 6; 7.5) mm. Apex of testis just posterior to oesophago-intestinal junction. Posterior end with four to three spiral coils. Tail 205–247 (224; 197) long, with conical lateral appendages; base of caudal appendages at 24–34 (29) from tail tip and 7–10 (8) long. In a single male of which electron micrographs were taken, the phasmid is distinct at the base of the lateral appendage (Figure 4C). A single postdeirid situated on left side, at 503–1070 (751) from tail tip (Figure 3B). Nine precloacal papillae; a single median papilla, slightly anterior to fourth precloacal pair, close to cloaca, and four subventral pairs (pairs 1–4). Six pairs of postcloacal papillae: five pairs anterior to pointed lateral caudal appendages (Figures 1I and 3B), a small single pair posterior to appendages; pairs 5–8 and pair 10 subventral, pair 9 in laterodorsal position; pair 6 larger than remaining papillae (Figures 1I and 3B). *Area rugosa* precloacal, beginning at 5.61–9.45 (8.20) mm from tail tip and ending approximately at level between second and third pairs of precloacal papillae; composed of longitudinal cuticular crests arranged on dog-bone-shaped transverse cuticular fields (Figure 3C); *area rugosa* vague towards its beginning and end. Spicules unequal. Right spicule short and robust, 103–138 (120; 139) long, with a pointed tip (Figures 1K and 3D). Left spicule weakly sclerotised, 280–302 (291; 345) long, with intricately looped, widened part close to peg-like distal end (Figure 3E, G); in several specimens a membranous sheath appears pushed backwards over the handle like a sleeve, in two specimens this sheath projects from the cloaca; Spicular ratio: 2.0–2.8 (2.5; 2.5).

*Female*: Body length 104–136 (120; 119) mm. Maximum body width at level oesophago-intestinal junction 589–785 (700; 797 maximum width). Cephalic elevations 73–115 (90; 100) apart in lateral view. Buccal capsule small and inconspicuous, 20–27 (25, *n* = 6) long and 21–26 (23, *n* = 6) wide in lateral view, 21–25 (23, *n* = 3) long and 17–27 (23, *n* = 3) wide in dorsoventral view. Nerve ring and deirids at 201–305 (244; 219) and 379–535 (449; 409 and 484) from anterior end, respectively. Muscular oesophagus 488–627 (546, *n* = 8) long; often bent and folded as result of muscle contraction. Glandular oesophagus 8.1–11.3 (9.9) mm long. Oesophagus total length 8.6–11.9 (10.5; 10.0) mm. Vulva a longitudinal oval slit at 394–619 (476; 531) from anterior end, at level of posterior half of muscular oesophagus or close to junction between muscular and glandular oesophagus. Vagina 312–457 (383) long, with short *vagina vera* and well-developed, posteriorly directed *vagina uterina* that forms a chamber that is joined by ovejector in its posterior third (Figure 2C). Tail, curved dorsally, 384–545 (483) long, with conical lateral appendages, tip of tail on conspicuous, bulbous swelling (Figures 1F and 3A, F); base of caudal appendages at 38–47 (43; 47) from tail tip and 9–12 (11) long. Phasmids not seen.

*Microfilariae* (Figure 2D): With left cephalic hook, body slender, tapering posteriorly, 177–193 (185, *n* = 12) long and 6 (*n* = 12) wide. Only possible remnants of sheath seen.

## Discussion

The measurements of specimens of *S. graberi* studied herein correspond closely to those given for “*Setaria graberi* n. sp.” in Shoho’s (personal communication, 2000) manuscript. In addition, Shoho refers to the deirids as being a “bifid spiny formation”, as seen in the present specimens. With reference to cephalic structures, Shoho states that the “peribuccal ring, surrounding the small round mouth, appears relatively smaller compared with the width of the anterior part of the body”, and describes it as “appearing as if drawn out dorsally and ventrally”; a description that fits the present specimens well. He also points out that the “more obtusely ending terminal end of the female, with distinctly rounded knob” readily distinguishes this species from, amongst others, *S. bicoronata*. The bulbous tail is a very conspicuous character in the females described herein.

Regarding the male tail, Shoho lists a “deirid-like formation on the left side at the level of the distal part of the retracted longer spicule”, four pairs of precloacal papillae, plus a median papilla situated close to the anterior border of the cloaca, as well as five pairs of postcloacal papillae, anterior to the “sharply pointed lateral appendages”, plus two smaller pairs of papillae posterior to the appendages. This corresponds well with the arrangement of the postdeirid and precloacal papillae in the present material, and with the illustration of *S. graberi* by Troncy et al. [11]. However, as in the latters’ illustration, the current specimens possess only six pairs of postcloacal papillae, one of which is situated posterior to the lateral appendages.

The illustrations of Troncy et al. [11] are so detailed and representative of the current material, especially regarding the peribuccal crown and cephalic elevations in lateral, dorsoventral as well as apical view, shape of the female tail and arrangement of caudal papillae in the male, that they are reproduced in this paper. A single exception is the deirids, which are illustrated as having a single tip (Figure 1D). Their bifid character is difficult to observe in light microscopy specimens, but readily visible using SEM. Neither Shoho nor Troncy et al. [11] comment on the male tail being spirally coiled, but they may have omitted this as a common feature in *Setaria* males, as well as other filarial worms.

In view of the distinct similarity between the current specimens and those described as *S. graberi* in Shoho’s unpublished manuscript and those illustrated as such by Troncy et al. [11], we conclude that the specimens described herein belong to *S. graberi*. This is supported by the pronounced differences between the present material and other *Setaria* species from the same host genus and/or *Setaria* species possessing bifid deirids (see below). It should also be noted that some of Shoho’s material was of the same geographic origin, South Africa, as the specimens studied herein. In the absence of any of the previously described specimens, voucher material for this species has been deposited in two curated collections.

Excepting *S. graberi*, a further six *Setaria* species parasitize members of the genus *Redunca* Smith (see below). Of these, *S. hornbyi* Boulenger, 1921, *S. labiatopapillosa* (Alessandrini, 1848) Railliet & Henry, 1911 and *S. lamyfortensis* Troncy, Graber & Thal, 1968 differ from *S. graberi* in the possession of deirids with a single tip [4, 11]. The configuration of the deirids of *S. boulengeri* is unknown, and specimens for re-examination could not be obtained. However, Yeh’s [14] description of the species differs from that of *S. graberi* in the distinctly raised peribuccal crown of *S. boulengeri* and an undivided dorsal as well as ventral elevation.

Similar to *S. graberi*, the deirids in *S. bicoronata* and *S. pillersi* Thwaite, 1927 are bifid [4], but the dorsal and ventral cephalic elevations of *S. pillersi* are square-shaped, undivided and spaced only 30–40 μm apart, as opposed to the bifid cephalic elevations in *S. graberi*, that are 71–93 μm apart in males and 73–115 μm in females.

*Setaria graberi* most closely resembles *S. bicoronata* in the presence of bifid deirids, but also in the configuration of the cephalic elevations in lateral and dorsoventral view. Examination of the latter structures in apical view, however, reveals distinct differences. While the apical view of the peribuccal crown together with the dorsal and ventral elevations portrays a more or less distinct rectangle in *S. bicoronata* [11], it is dumbbell-shaped in *S. graberi*. Also, in lateral view, the outer border of the dorsal and ventral elevations extends further posteriad on the head of *S. bicoronata* than in *S. graberi*, where the elevations appear perched on the anterior part of the apex (Figures 1A and 2A). In addition, the conspicuous bulbous swelling of the female tail sets *S. graberi* apart not only from *S. bicoronata*, but from all other *Setaria* species in African artiodactylids.

A further two *Setaria* species with bifid deirids have been described from hosts other than *Redunca*, *S. saegeri* (Le Van Hoa, 1961) from *Sylvicapra grimmia* Linnaeus in the Congo and *S. kabargi* Kadenazii, 1948 from *Moscus moschiferus* Linnaeus in Central Asia [4, 13]. However, both species are readily distinguished from *S. graberi* by the shape of their flat-topped cephalic elevations [4, 13].

The following *Setaria* species have been recorded from hosts of the genus *Redunca*, including *R. arundinum*, Mountain reedbuck *R. fulvorufula* (Afzelius) and *R. redunca*, in Africa:

1. ***Setaria bicoronata*** (Linstow, 1901) Railliet & Henry, 1911 from *R. arundinum* in Malawi, Mozambique and Zambia [14], the Democratic Republic of the Congo [4] and Zimbabwe [3].
2. ***Setaria boulengeri*** Thwaite, 1927 from *R. arundinum* and *R. fulvorufula* in South Africa [7, 10], and from *R. redunca* in Tanzania [9].
3. ***Setaria graberi*** Shoho in Troncy, Graber & Thal, 1976 from *R. arundinum* in South Africa (Shoho, personal communication 2000, this study) and from *R. redunca* in Tanzania [9], the Central African Republic and Republic of Chad [11].
4. ***Setaria hornbyi*** Boulenger, 1921 from *R. arundinum* in South Africa [6].
5. ***Setaria labiatopapillosa*** (Allessandrini, 1848) Railliet & Henry, 1911 from *R. arundinum* in South Africa [1, 12].
6. ***Setaria lamyfortensis*** Troncy, Graber & Thal, 1968 from *R. redunca* in the Central African Republic and Republic of Chad [11].
7. ***Setaria pillersi*** Thwaite, 1927 in *R. arundinum* from the Democratic Republic of the Congo [4] and in *R. redunca* from the Republic of Chad [11].
8. ***Setaria* sp.** in *R. arundinum* in Zimbabwe [8] and South Africa [1].
